# Human Ideals in Industry

**Published:** 1921-05-21

**Authors:** 


### Human Ideals in Industry.

It would be an irreparable misfortune to this country if anything should impede the contribution to social progress represented in the rise of organised labour during the last eighty years, says the *Journal of Industrial Welfare*. If it be true that the old form of Trades Unionism has suffered a defeat, and that the employers have been victorious, "then our prayers should be with the latter, for more depends on their conduct of the crisis now than they have dreamed of. Never before have they had such an opportunity for demonstrating their honest purpose and magnanimity. Never before has the ground been so clear for industrial reconstruction." This is perhaps putting the employers' opportunity rather high—for there can only be "reconstruction" with the goodwill of both sides—but our contemporary is wisely inspired in its broad analysis of the elements of a problem on the solution of which our entire social fabric depends.

Briefly, there are three aspects of industry that need emphasis. First there must be more efficiency in industrial management. All the forces of science must be concentrated on the tasks of relieving human toil, of protecting the workers from unnecessary hazards, and of turning their labour to the highest advantage. Secondly, there must be a definite recognition of the human factor as a fundamental problem of industry, and a determination to deal with it separately and adequately, with no less ardour than is given to the care of the machinery and structure; but, thirdly, industry must be realised as one of the highest forms of service, and not as a gigantic Monte Carlo for the speculator. Mr. Robert R. Hyde, the well-known authority on the welfare of the worker, puts the matter in another way and with even more emphasis when he says that the future of industry in this country depends not so much upon our mechanical efficiency as upon the way in which the human material is handled.

Moreover, the effect on the health of the worker, the point of view with which in this section of The Hospital we are more expressly concerned, is so marked in those establishments in which welfare work is seriously carried on that there can be no two opinions about its permanent value to the nation. We regret to notice that certain firms, looking about for possibilities of saving costs during the period of extreme trade depression, have begun by economising in welfare work. It is almost as if the head of a family, faced with a reduction of income, started first to cut down the family bread bill.

